# Viruses in Cancers of the Digestive System: Active Contributors or Idle Bystanders?

**DOI:** 10.3390/ijms21218133

**Published:** 2020-10-30

**Authors:** Martin Marônek, René Link, Giovanni Monteleone, Roman Gardlík, Carmine Stolfi

**Affiliations:** 1 Institute of Molecular Biomedicine, Faculty of Medicine, Comenius University, 811 08 Bratislava, Slovakia; martin.maronek@gmail.com (M.M.); romangardlik@gmail.com (R.G.); 2 Institute of Experimental Medicine, Faculty of Medicine, University of Pavol Jozef Šafárik, 040 11 Košice, Slovakia; rlink4@gmail.com; 3 Department of Systems Medicine, University of Rome “Tor Vergata”, 00133 Rome, Italy; gi.monteleone@med.uniroma2.it; 4 Division of Clinical Biochemistry and Clinical Molecular Biology, University of Rome “Tor Vergata”, 00133 Rome, Italy

**Keywords:** virome, phages, eukaryotic viruses, colorectal cancer, HBV, HCV, HPV, EBV, p53, Wnt/β-catenin

## Abstract

The human virome, which is a collection of all the viruses that are present in the human body, is increasingly being recognized as an essential part of the human microbiota. The human gastrointestinal tract and related organs (e.g., liver, pancreas, and gallbladder)—composing the gastrointestinal (or digestive) system—contain a huge number of viral particles which contribute to maintaining tissue homeostasis and keeping our body healthy. However, perturbations of the virome steady-state may, both directly and indirectly, ignite/sustain oncogenic mechanisms contributing to the initiation of a dysplastic process and/or cancer progression. In this review, we summarize and discuss the available evidence on the association and role of viruses in the development of cancers of the digestive system.

## 1. Introduction

The human microbiota represents one of the most complex ecosystems on Earth, spanning bacteria, archaea, fungi, and viruses. Such microbes have coevolved with humans for millions of years, influencing our development and shaping, in particular, our immune defenses [1]. Despite this, for many years, there was modest interest in the microbiota among researchers and it is only in the last decade that it has become a major research area. Nowadays, the microbiota is considered to be crucial for our immune, hormonal, and metabolic homeostasis, and perturbations in its composition contribute to the emergence of various diseases, such as inflammatory bowel diseases, obesity, and metabolic syndrome [2].

The human virome, which is a collection of all the viruses that are present in the human body [3], is increasingly being recognized as an essential part of the human microbiota and emerging as a new and interesting field of study due to recent advances in the detection and characterization of the viral genome within the background of the ample human genome. The virome composition changes within different organs/tissues, as each compartment of the body is characterized by a distinctive microenvironment. In addition, the viral communities are influenced by both endogenous and exogenous factors, such as age, diet, and the presence of other components of the microbiota [4,5].

The human gastrointestinal tract and related organs (e.g., liver, pancreas, and gallbladder)—composing the gastrointestinal (or digestive) system—contain a huge number of viral particles (estimated to be more than 10^15^) [6,7], which contribute to maintaining tissue homeostasis and keeping our body healthy [8]. However, virome steady-state perturbations may ignite different diseases, such as hepatitis and inflammatory bowel diseases (IBDs), and recent research is shedding light on the role of viral entities in promoting dysplasia and ultimately, cancer.

In this review, we summarize and discuss the available evidence on the association and role of viruses in the initiation and development of cancers of the gastrointestinal system.

## 2. Human Virome: Main Players

A virome includes all of the nucleic acids (single-stranded DNA, double-stranded DNA, single-stranded RNA, and double-stranded RNA) belonging to the viruses associated with a particular ecosystem [9]. Such viruses can generally be distinguished based on the host preference, with virtually any form of life potentially acting as a host. The human gut virome is established soon after birth and comprises a diverse collection of viral particles that infect both prokaryotic and eukaryotic cells [3]. Research has mainly focused on two groups of viruses: bacteriophages (or phages), which infect bacteria and overwhelm the other classes in terms of abundance, and eukaryotic viruses (hereinafter referred to as viruses), which infect our own cells. Although their importance cannot be underestimated, other components of the gut virome, such as viruses that infect protozoans, fungi, archaea, and even other viruses, are not as deeply studied as the above-mentioned groups due to a limited understanding of their function within the environment of the human body [10]. By either directly affecting host cell behavior/viability or by preying upon certain species of bacteria, with the ultimate result of altering bacterial communities, enteric viruses contribute to the homeostasis of the digestive apparatus. For instance, it has been shown that intestinal abnormalities of germ-free mice were alleviated following infection of the animals with the murine norovirus strain CR6 [11]. Moreover, the pretreatment of mice with antiviral drugs resulted in more severe colitis in a mouse model of dextran sulphate sodium-induced colitis [12]. On the other hand, some members of the gut virome may contribute, either directly or indirectly, such as via increasing the frequency of mutations, chromosomal rearrangements, and insertional mutagenesis, to the emergence and development of cancers [13].

### 2.1. Phages

As bacterial predators, phages extensively contribute to the abundance and composition of the gut microbiota and have been mainly studied for their impact on intestinal inflammation, as in the case of Crohn’s disease and ulcerative colitis [14,15]. To date, several studies have suggested that phages may be an active driving force of gut carcinogenesis [16]. One possibility in this regard is that they act via modulation of the local microenvironment of the alimentary canal in a fashion that promotes the invasion and proliferation of oncogenic bacteria [16]. One opportunistic bacteria which has already been implicated in gastrointestinal cancer is *Fusobacterium nucleatum* [17]. Alternatively, phages may be immunogenic, as a high presence of phage DNA has been shown to aggravate ulcerative colitis via the engagement of Toll-like receptors (TLRs) and interferon-γ production [18]. Phages can employ two different cycles to infect and modulate the bacterial population—lytic and lysogenic—and can even switch between them on certain occasions [19]. In the lytic cycle, the infection of bacteria results in destruction of the infected cell and the subsequent release of viral progeny. In the lysogenic cycle, the genomic material of the phage randomly integrates into the host genome and remains there as a prophage replicating along with it. This process, known as horizontal gene transfer, provides bacteria with additional genes [20]. Such genes may code toxins and/or products, facilitating, among other things, bacterial mucosal adhesion, evasion from the host immune system, resistance to antibiotics, and/or the invading ability [21,22,23]. Such transformed bacteria could potentially cause tissue damage, inflammation, and progression toward carcinogenesis. On the other hand, tumor-specific phage particles have been reported to promote tumor regression/destruction in mouse melanoma models [24,25] and phage therapy has even been considered as a plausible treatment option for human cancer [26]. In this context, protein–protein interaction between GP24 of T4 and HAP1 phages and integrin β3 or the HSP90 receptor of cancer cells was shown to inhibit metastasis in melanoma and lung cancer in murine (B16 and LLC) and human (HS294T and A549) cell lines [27,28]. Interestingly, under certain circumstances, the interaction between phages and human cells may be important for phage infection. Shan et al. reported that phage phiCDHS1 killed the pathogen *Clostridium difficile* faster when placed in a culture of human colon cancer cells [29]. Furthermore, this interaction appeared to be cell-specific, since HeLa cells did not confer the observed effect [29]. Overall, it remains to be determined which interactions and under which circumstances phages are potentially harmful or protective in the development of gastrointestinal cancer.

### 2.2. Viruses

Viruses can infect all the cells of our body and other eukaryotic organisms colonizing our digestive tract. The diversity of such viruses is commonly evaluated by determining the viral families or species in a given sample. This is usually expressed as the number of reads as a surrogate of their relative abundance. Among the DNA viruses, some can establish persistent infections, such as those belonging to the Herpesviridae family, whereas others, such as Anelloviruses, have not been associated with a specific pathology [30]. In contrast, RNA viruses seem to cause acute infections. However, as the majority of metagenomic studies have focused on DNA viruses, it is possible that RNA virus families are also widely distributed in the human body. Persistent viruses are of particular importance since they can establish a long-term relationship with their host and influence the inflammatory status of the body. Such a persistent inflammatory condition can promote, in some cases, a carcinogenic milieu that favors the genesis and development of cancer cells [31,32]. Tumor viruses acting by shaping the immune system towards the production of carcinogenic and/or immune-suppressive cytokines/factors are referred to as indirect carcinogens [33]. On the other hand, viruses that are capable of incorporating their genetic information into the human DNA—a process that can result in the dysregulation of oncogenes and/or inactivation of tumor suppressive genes—are referred to as direct carcinogens [33]. However, it is important to point out that direct and indirect mechanisms of virus-driven carcinogenesis are not mutually exclusive and some tissues may be equally dependent on both mechanisms for oncogenic transformation, such as the liver and stomach [13]. While current evidence strongly suggests a role of certain viruses, e.g., Epstein-Barr virus (EBV) in the pathogenesis of lymphoma in IBD patients [34] or human papillomavirus (HPV) in cervical cancer [35], the role of these and other viruses in gastrointestinal cancer remains less clear.

## 3. Role of Viruses in the Development of Cancers of the Gastrointestinal System

In the last two decades, researchers have attempted to unravel the potential link between virus infection and cancer development. While most of the studies in this regard only aimed at detecting the presence of viral particles in cancer patients, some aimed to shed light on the interaction/causality between putative tumor viruses and transformed cells. The associations of specific viruses with cancers of the gastrointestinal tract and related organs, as well as the virus-driven molecular mechanisms (potentially contributing to the emergence/development of such malignancies), are summarized in Table 1 and discussed below.

### 3.1. Oral Cancer

Compared to other cancers of the digestive system, oral cancer (OC) has a lower worldwide incidence [66]. However, this statistic does not mean that less attention should be directed toward it. The oral cavity comes into contact with food on a daily basis, which is a source of bacteria and viruses. Saliva helps digest the food, moistens it to favor the passage into the stomach, and acts as a protective layer for the surrounding tissue. Many OC cases are attributed to smoking and dietary habits [67]. In this context, the consumption of betel quid, which contains areca nut and tobacco, is believed to be partially responsible for the higher occurrence of cases of oral submucous fibrosis and OC in the Indian population compared with people from other countries [68]. Certain viruses, such as HPV, have been suggested to play a role in the development of OC. In 1992, a study by Maden and coworkers reported a 2.9-fold and 6.2-fold higher risk of developing OC for men who were positive for HPV-6 and HPV-16, respectively [36]. Since then, HPV has been suggested to account for as much as 30% of all OC cases [69]. However, there is considerable variability in the results of HPV prevalence in the published literature. For instance, Kouvousi et al. found that the HPV prevalence in OC samples is 11% [70]. In the same paper, the mRNA expression of HPV E6 and E7 genes, which are known protooncogenes, was reported in almost 9% of the cases [70]. On the other hand, HPV prevalence as high as 65% has been reported in OC patients in a Swedish study [37]. Accumulating evidence seems to indicate that OC-associated viral infections are linked to certain geographical areas where a virus reaches a higher prevalence. In a study by Jalouli et al., the prevalence of HPV, EBV, and herpes simplex virus (HSV) was compared in OC samples [37]. When considering all of the samples, 55%, 35%, and 15% of them were positive for EBV, HPV, and HSV, respectively. HPV was most abundant in Sudan, reaching a prevalence of 65%, whereas both EBV and HSV were most prevalent in the United Kingdom (with values of 80% and 55%, respectively). Of note, single infections with HSV and EBV, as well as co-infections with the same viruses, occurred at a significantly higher proportion in samples taken from patients inhabiting industrialized countries (e.g., Sweden, Norway, the United Kingdom, and the USA) compared with samples taken from patients living in developing countries (e.g., Sudan, India, Sri Lanka, and Yemen) [37]. Data indicating a probable role of HPV in the rise of OC were obtained by González-Ramírez and colleagues, who found a two-times higher presence of HPV DNA in the Mexican population with a diagnosis of OC compared to controls (5% vs. 2.5%, respectively). It is noteworthy that OC patients presented no additional risk factors, such as alcohol consumption or smoking [71]. Subsequently, a meta-analysis performed in India reported HPV positivity in almost 40% of patients bearing OC—with an odds ratio of 2.82—thus indicating a significantly higher risk of developing OC for HPV-positive patients compared with the general population [72]. None of the standard clinicopathological variables (e.g., age, sex, smoking and oral tobacco history, grade, or site) were predictive for HPV positivity [73].

Interestingly, the percentage of HPV-positive OC patients without any history of oral tobacco use was more than 31%, whereas only 10% of OC patients with previous oral tobacco use tested positive for HPV infection [73].

Mechanistically, recent research has been focusing on elucidating potential viral interactions with oncogenic signaling pathways. For instance, the overexpression of p16—a tumor suppressor protein—has been detected in OC samples and suggested to act as a possible surrogate marker for infections with high-risk HPV strains [74]. Several reports have highlighted the importance of p53 in HPV infections with regards to p53 mutations [75] or the p53 expression status [76], or as an association with poor patient survival [77]. Taken together, although causative evidence remains elusive, the above studies indicate a strong association between HPV infection and a higher risk of developing OC.

### 3.2. Esophageal Cancer

Esophageal cancer (EC) usually manifests as either esophageal adenocarcinoma (EAC) or esophageal squamous cell carcinoma (ESCC) [78]. The notion that HPV may be involved in EC arose when its presence was discovered in specimens taken from patients with this neoplasia [79]. However, there is no clear evidence to date indicating that the presence of HPV represents a risk factor for the development of EC, as reports are controversial. In 1997, a prospective study by Bjørge et al. found an increased risk of developing EC among HPV-16-positive subjects in Norway [38]. Conversely, two years later, a Swedish nationwide case-control study of HPV-16 and HPV-18 did not report any association between HPV and either EAC or ESCC, even when adjusted for additional factors such as smoking or alcohol consumption [80]. In line with this latter evidence, the occurrence of HPV (both high- and low-risk phenotypes) did not differ between ESCC and control samples in Poland [81]. Observational studies from China declared that HPV infection is independent of region and ethnic group [40] and range from stating that HPV infection may be a risk factor [39] to suggesting that HPV infection has a causative role in the development of EC [82]. Other regional studies from Iran [83], Turkey and Somalia [84], Colombia and Chile [85], Brazil [86], and Australia [87] confirmed the presence of HPV in either EC or both controls and EC samples. Adding to the regional inconsistency, one study from Brazil did not find any HPV-positive ESCC samples [88] and another identified only a few HPV-infected samples in Australia [89]. In a study by Chang et al., the analysis of cytomegalovirus (CMV) and HSV was included, in addition to HPV and EBV. However, only HPV positivity was found in all of the carcinomas [90]. In a Chinese cohort, Xi et al. reported a significantly higher HPV16 E6 expression in ESCC patients compared to controls. In the former, a positive HPV16 infection status negatively correlated with 3- and 5-year overall survival, as well as with progression-free survival [91]. Given such data, the authors even suggested that HPV infection may represent a marker for predicting the prognosis of ESCC patients. Taken together, the above reports indicate a higher prevalence of HPV in EC specimens than in controls, which may suggest a certain role of HPV in the development of EC. However, the causality of this observation has not yet been elucidated. Concerning the role of EBV in EC, most studies agree that EBV infection is not involved in or does not correlate with the development of EAC and ESCC [92,93,94,95,96,97], although a German study by Awerkiew et al. reported that EC was associated with EBV, but not with HPV [46].

Although the mechanisms of conversion of normal squamous cells to carcinoma cells have not been completely elucidated, some studies have highlighted the role of several viruses in such an event. For instance, it has been found that the *HPV E6* and *HPV E7* genes contribute to ESCC pathogenesis via DNA demethylation and upregulation of the human leukocyte antigen (HLA)-DQB1 [98]. Moreover, HPV-16 E6 downregulated microRNA (miR)-125b—a negative regulator of the Wnt/β-catenin signaling pathway—thus contributing to EC carcinogenesis [99,100]. Besides the above-mentioned effects, HPV is likely involved in aberrations of the p53 protein and retinoblastoma pathway [101].

Some ESCC cases may arise as a combination of genetic susceptibility and HPV infection. Yang et al. managed to identify two risk loci, which were found to not only increase the risk for ESCC, but also interact with the HPV serological status [102]. Furthermore, in combination with HPV16 seropositivity, two p53 single nucleotide polymorphisms Arg/Arg and Arg/Pro increased the risk for ESCC [103]. Interestingly, HPV infection may not always worsen the prognosis of ESCC, as it has been found that HPV increased the tumor response to chemoradiation [104]. However, data are not clear regarding p53, as it was reported that HPV infection and p53 expression were not of any prognostic value [105]. Finally, HPV infection has been associated with telomere length. Zhang et al. reported that HPV-infected ESCC tumor tissue contained longer telomeres, due to the DNA methylation status of telomerase reverse transcriptase, compared with HPV-negative tissue [106]. 

Despite all of the data presented, two issues must be stressed. Firstly, most of the studies were observational (either prognostic or retrospective) and thus lack causality. Secondly, all of the studies were designed to detect the presence of either HPV or EBV in EC samples and, to the best of our knowledge, there is no study analyzing a complete virome panel in EC. Therefore, the answer to the question of whether viruses lead to a higher risk of EC may be even more complex than previously thought.

### 3.3. Gastric Cancer

Data from epidemiological studies performed across the globe do not unambiguously unravel the potential role of HPV in the pathogenesis of gastric cancer (GC) [107,108]. Data on EBV and GC are also controversial. EBV is estimated to be widespread and studies carried out in the United Kingdom and United States reported an EBV seroprevalence higher than 90% in specific subgroups of people [109,110]. Despite that, EBV was found in only 9% of all GCs worldwide [111]. Similarly, the abundance of EBV was found to be very low in a Chinese population with GC incidence [112]. Yuan et al. did not detect any HPV or EBV DNA in GC tissue samples, whereas de Souza et al. found that only 20% of the tumors were positive for EBV and observed no expression of the HPV E6 and E7 oncogenic proteins [113,114]. In contrast, Martínez-López and co-workers revealed the presence of EBV in GC specimens and proposed that EBV may exert carcinogenic actions, either directly, by infecting epithelial cells, or indirectly, by inducing inflammation [47]. The variability of the above findings is further enhanced by the results of Corallo et al. [48]. The authors showed that EBV-positive metastatic GC patients, who did not receive immune checkpoint inhibitors, had a long-lasting and complete response to first-line chemotherapy, as well as better survival, compared to metastatic GC patients who were EBV-negative [48]. 

Recently, a number of studies have tried to shed light on the molecular mechanisms underlying the effects of EBV on GC pathogenesis. Zhang et al. [115] focused on the expression of the LMP2A protein, which is one of the proteins usually expressed by EBV in infected cells, and human epidermal growth factor receptor 2 (HER2), which is a protooncogen that has been associated with the development of different cancer types, such as esophageal [116], gastric [117], and breast [118] cancer. The overexpression of LMP2A in EBV-negative cells led to a decrease of HER2 mRNA. Interestingly, EBV-associated GC cases exhibited the highest survival when compared with cases bearing either low concentrations of LMP2A and HER2 or low concentrations of LMP2A and high concentrations of HER2 [115]. Along the same line is the study by Constanza Camargo et al., showing a higher rate of survival of EBV-positive GC patients compared with EBV-negative ones [119]. In contrast, the results published by Wang et al. seem to contradict the supposed beneficial effect of EBV in GC [120]. Besides proteins, EBV encodes many miRNAs and, among these, EBV-miR-BART3-3p was reported to promote the growth of GC cells, both in vitro and in vivo, by inhibiting their senescence induced by an oncogene (RASG12V) or chemotherapy (irinotecan) [120]. In conclusion, although EBV positivity seems to confer some sort of protection against GC, it is important to point out that the sole presence or absence of EBV in GC samples does not imply a direct causality or a role of EBV in this neoplasia. Epidemiological studies carried out in other countries and aimed at assessing whether EBV is latent or active in patients who develop GC would help clarify this issue.

### 3.4. Liver Cancer

In 2012, 770,000 cases of liver cancer occurred worldwide, of which 430,000 (56%) and 150,000 (20%) were attributed to hepatitis B virus (HBV) and hepatitis C virus (HCV), respectively [121]. Most of the liver cancer cases in the Western world are attributable to HCV, whereas HBV represents the major driver of liver carcinogenesis in developing countries [121]. The main non-viral risk factors for liver cancer are presumed to be alcohol consumption, tobacco smoking, diabetes, obesity, and aflatoxin B1 exposure in Africa and Eastern Asia [122]. The most common type of liver cancer is hepatocellular carcinoma (HCC), although in certain parts of the globe, such as South-Eastern Asia, intrahepatic bile duct cancer is the more frequent liver-associated neoplasia, due to the high presence of fluke infections [123]. Due to the huge amount of data on the proposed mechanisms by which HBV and HCV increase the risk of developing HCC, for detailed information on this topic, we refer the reader to other more specific reviews [124,125].

Although the above-mentioned viruses and factors account for the majority of HCC cases, other viruses, such as HPV and EBV, may play a role in liver carcinogenesis. So far, a very limited number of studies have focused on elucidating the potential role of HPV in HCC. A study dated to 1992 found HCC samples infected with either HPV-16 or HPV-18 and suggested that HPV could act synergistically with HBV to promote HCC development [126]. However, to the best of our knowledge, these results have not been replicated and more studies are needed to ascertain the possibility of such viral cooperation. Sugawara et al. detected EBV in one third of HCC samples [127], although subsequent studies did not confirm any involvement of EBV in the development of HCC [128,129,130,131]. Similarly, in a study from China, the authors found that more than 28% of HCC samples were EBV-positive and argued for a possible association between EBV and the genesis and development of HCC [49]. However, mechanistic studies demonstrating a clear cause–effect of the above-mentioned hypothesis are lacking. Song and colleagues associated the expression of the EBV-encoded gene 3 (EBI3), which has been found to be upregulated in a variety of cancers, e.g., lung [132], pancreatic [133], or cervical [134], with the outcome of HCC patients after curative resection [135]. A low expression of EBI3 was associated with recurrence of the disease and shorter survival [135]. Interestingly, this result is in line with data reporting a higher survival of GC patients who were EBV-positive, compared with EBV-negative ones [119]. Although the above-mentioned evidence would suggest that EBV confers some kind of protection to liver cancer patients, this is not true for all cases. Indeed, in a distinct subset of HCC characterized by an immune cell stroma, EBV-positive tumor-infiltrating leukocytes were associated with a worse prognosis [136]. 

The TT virus (TTV), first isolated in 1997 from a Japanese patient and named after his initials [137], is another virus suggested to play a role in HCC pathogenesis, although early studies from different countries did not report any association between either the presence of TTV and development of HCC [138,139] or the severity or prognosis of TTV-positive versus -negative HCC patients [140]. In 2002, Tokita et al. indicated a high TTV load as an independent risk factor for HCC [55]. Conversely, in Egyptian patients, TTV was found to be abundant in, but not associated with, HBV, HCV, or HCC [141]. To date, the evidence produced does not seem to support an association between the presence of TTV and an increased risk of developing HCC. Further studies are needed in order to clarify this issue.

### 3.5. Bile Duct Cancer

Intrahepatic (IHCC) and extrahepatic cholangiocarcinoma (EHCC) represent the two most common forms of bile duct cancer [142]. There are a small number of studies investigating the relation between viruses and cholangiocarcinoma and mainly rely on an indirect approach, focused on the survival and prognosis of HBV- and/or HCV-infected patients. In this context, some potential mechanisms linking HCV and cholangiocarcinoma have been discussed by Navas et al. [143]. A study by Zhou et al. reported an odds ratio of 5.39 and 2.6 for HBV and HCV infection in IHCC, respectively [57]. Other studies are in agreement, stating that hepatitis viruses may be possible risk factors in the pathogenesis in cholangiocarcinoma [144,145,146]. A recent meta-analysis by Tan et al. concluded that both HBV and HCV infections significantly increase the risk of developing both IHCC and EHCC [147]. Nevertheless, some reports have failed to provide evidence linking HBV and HCV to bile duct carcinogenesis [148,149]. Studies analyzing the survival of cholangiocarcinoma patients with or without HBV infection are also not in agreement [150,151]. More recently, a study by Klufah and colleagues found a 59% prevalence of human polyomaviruses in cholangiocarcinoma patients [152]. The above results suggest the need for further investigations, in order to figure out the role of viruses in the pathogenesis of bile duct cancer.

### 3.6. Pancreatic Cancer

Based on the available literature, a limited number of viruses are supposedly involved in the development of pancreatic cancer (PC), with HBV and HCV as the most likely contributors. The available studies/meta-analyses do not clearly prove whether HBV and HCV infections are associated with a higher risk of developing PC. A meta-analysis by Wang et al. suggested that HBV infection may increase the risk of PC [153]. In contrast, Abe and co-workers found no association between HBV or HCV infection and PC [154]. Other papers have presented conflicting results. While no association has been reported in studies carried out in Southern China and Taiwan [155,156], a nationwide study from Sweden pointed towards a higher risk of PC in the HBV cohort [157]. However, it has to be pointed out that this latter observation did not reach statistical significance. In accordance with the Swedish study was the REVEAL-HBV cohort study by Iloeje et al. [56]. By investigating the presence of HBV in pancreatic tissue specimens, the authors suggested that chronic HBV infection could be associated with an increased risk of PC [56]. Research focusing on past HBV and HCV exposure is also contradictory. Indeed, some studies concluded that an association between past HBV infection and the risk of developing PC could be possible [58,158], whereas Tang et al. reported no such an association [159]. Interestingly, in a study comparing the risk of HCC and PC development in HBV-infected individuals, the authors reported no increased risk for PC, but a 17-fold higher risk for HCC [160]. Research investigating the mechanism/s by which some viruses might increase the risk of PC is rather scarce. In a study performed on the pancreatic cancer cell lines PANC-1 and SW1990, a higher expression of receptor tyrosine kinase ErbB4 and TGF-α was found after HBV infection, along with the upregulation of MAPK and PI3K/AKT pathways [161]. To the best of our knowledge, no evidence advocates for a role of EBV in PC. In a study by Tomasiewicz et al., TT virus DNA was found in the sera of two patients who developed PC [162]. However, as we are not aware of any other report evaluating the association between TT virus and PC, it is impossible to draw any conclusion.

Taken together, despite certain advances that shed some light on the role of hepatitis viruses in the development of PC, more research is needed to uncover the putative molecular mechanisms governing the pancreatic carcinogenesis associated with viral infections.

### 3.7. Colorectal Cancer

Colorectal cancer (CRC), defined as a cancer arising in the human colon and/or rectum, is one of the most common and studied types of malignancy. In 2018, CRC was the third most frequently diagnosed cancer (with more than 1.8 million cases) and the second in terms of mortality, accounting for almost 900,000 deaths worldwide [66]. The human colon contains a vast viral load, comprising both viruses and phages. It is well-accepted that viruses help maintain proper functioning of the intestine [163] and that a shift of the composition and/or abundance of the intestinal virome far from the physiologic state may predispose to dysplasia and ultimately, cancer [164]. Although implications for viral engagement in the development of CRC are growing, current evidence is still relatively scarce. Given the sheer amount of living organisms and viruses in the colon, it is a great challenge to pinpoint the role of a single viral strain in the process of colon carcinogenesis. Therefore, it is not surprising that many studies have found associations which need to be further tested and verified.

#### 3.7.1. HBV and HCV

In contrast to their role in promoting liver carcinogenesis, hepatitis viruses (i.e., HBV and HCV) seem to decrease the risk of colorectal liver metastasis in CRC patients [165,166,167]. In line with this assumption, Li Destri and colleagues found better 5-year disease free survival and a lower incidence of metachronous liver metastases in hepatitis virus-infected CRC patients [168], whereas a subsequent study found no correlation between HCV prevalence and subjects symptomatic for CRC [169]. More recently, Su et al. suggested HBV infection to be significantly associated with an increased risk for CRC [59]. The results of this latter study oppose the putative protective effect of hepatitis virus infection against the development of liver metastases in CRC and highlight the need for additional research, in order to figure out such inconsistency.

#### 3.7.2. HPV

The first association between HPV infection and CRC is dated to 1990, when, by immunohistochemistry and in situ hybridization, Kirgan and co-workers found that the HPV antigen was present in 60% of benign tumors and 97% of carcinomas, compared with 23% of normal colon specimens [41]. The authors also detected the HPV genome in 27% of benign tumors and in nearly 43% of all carcinomas tested. The same research group subsequently confirmed such findings by PCR and Southern blotting [170]. In another study, HPV-18 DNA was detected in 53% of normal mucosal samples and 84% of CRC specimens [42]. Interestingly, Damin et al. found a similar abundance of another HPV strain (i.e., HPV-16) in CRC samples, whereas HPV-16 DNA was not detectable in the control group. However, no correlation between HPV presence and prognostic predictors could be observed [171]. Along the same lines is a study by Giuliani and colleagues, reporting the presence of HPV DNA in 22 out of 66 (33.3%) CRC samples and indicating a possible association between HPV occurrence and an increased risk of developing CRC [172]. The possible contribution of HPV to CRC carcinogenesis was, however, questioned by other research articles which either did not find any presence [173] or only a low abundance [174,175] of HPV DNA in tumor specimens taken from CRC patients. Moreover, it has even been suggested that HPV-positivity might be caused by contamination from the anal canal or during sample processing [174]. Despite having identified only six out of 140 total CRC samples as HPV-positive compared to no positive samples in the control group, Mahmoudvand et al. still proposed a potential role for HPV in CRC carcinogenesis [176].

**Figure 1:**
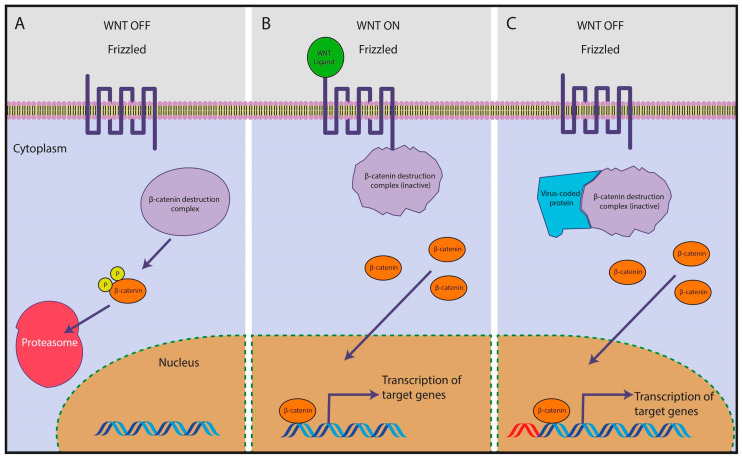
Virus-mediated modulation of the Wnt/β-catenin pathway. (**A**) When Wnt signalization is off, β-catenin is phosphorylated via the β-catenin destruction complex and subsequently degraded by the proteasome. (**B**) When a Wnt ligand binds a Wnt receptor (Frizzled), the β-catenin destruction complex is inactive, and β-catenin accumulates and enters the nucleus, where it modulates the transcription of target genes. (**C**) When infecting a cell, a virus may interact with some of the Wnt pathway components (e.g., the β-catenin destruction complex), leading to the accumulation of β-catenin and persistent transcription of its target genes, even in the absence of a Wnt ligand.

Several studies have attempted to uncover the mechanisms underlying the possible link between HPV infection and CRC. In this regard, Qiu et al. performed a gene expression analysis and found four upregulated and differentially expressed genes in HPV-positive CRC samples compared to HPV-negative tissues [177]. These genes coded proteins, namely WNT-5A, c-Myc, matrix metalloproteinase 7 (MMP-7), and AXIN2, which have been previously implicated in CRC pathogenesis [177]. For instance, the WNT-5A protein is a member of the WNT gene family, which is involved in key cellular processes, such as differentiation, proliferation, and migration via Wnt/β-catenin signalization [178]. In theory, once inserted into the genome of the cell, certain viruses such as HPV can interact with the Wnt pathway to promote the transcription of genes involved in proliferation (Figure 1).

In a similar fashion, c-Myc acts as a protooncogene which has been shown to be constitutively expressed, besides CRC [179], in other cancer types, such as breast cancer [180] and lung cancer [181,182]. MMP-7 has been associated with colorectal tumor growth and metastasis and one meta-analysis found that its overexpression could even serve as a prognostic factor [183]. Likewise, AXIN2 has been associated with CRC [184].

Another study reported a correlation between HPV infection and worse clinical stages of CRC [185]. Notably, HPV infection also correlated with higher activity of the transcription factor STAT3 and a higher expression of the pro-inflammatory cytokine interleukin-17 [185], both of which have been shown to be involved in CRC carcinogenesis [32]. The ability to evade or suppress programmed cell death is one of the hallmarks of cancer cells [186]. In this context, Karbasi et al. found a significantly lower expression of the pro-apoptotic genes FAS and DR5 in HPV-positive CRC samples compared to normal tissue [187]. Other signaling pathways, such as the MAPK/ERK pathway, may be involved, as Buyru et al. observed that 56% of HPV-positive CRC samples carried mutations in the protooncogene *K-Ras* [188]. Although it remains inconclusive, research has also been conducted to determine a possible association between HPV infection and p53 mutation [189,190].

Taken together, despite a considerable amount of data pointing toward the notion of a carcinogenic effect of HPV infection, more studies are needed to elucidate whether such infection arises before, during, or after CRC development and whether the aforementioned changes in gene expression are strictly dependent on HPV, rather than driven by other factors.

#### 3.7.3. EBV

Studies concerning the association of EBV with CRC are limited. One of the first reports on the potential carcinogenic effect of EBV comes from Song and colleagues, who detected the presence of EBV in almost 80% of CRC specimens [50]. Next, Fiorina et al. reported the presence of EBV DNA in more than half of the CRC tumor samples [51]. However, due to the huge variability in the amount of EBV DNA detected, the authors concluded that EBV was not significantly involved in CRC [51]. A later study by Sarvari and co-workers seemed to sustain this conclusion, as the authors could only detect EBV in 1 out of 70 tumor specimens taken from Iranian CRC patients [191]. It needs, however, to be pointed out that, as the prevalence of EBV infection varies considerably worldwide, the scope of such population studies may be limited. As a result, the absence of EBV infection may not necessarily mean that EBV does not cause or increase the risk for CRC development. Recently, Malki and colleagues investigated the role of HPV-EBV co-infection in CRC [192]. Indeed, whilst each of these viruses does not seem to present an immediate threat when acting alone, they could pose a much greater challenge to the colon when occurring together. HPV-EBV co-infection was reported in 17% of the samples and associated with invasive carcinoma features [192]. However, another study by the same group partially contradicted this conclusion, as no significant association between the co-presence of high-risk HPV and EBV and clinicopathological variables was found [193]. Finally, Liang et al. investigated the potential effect of EBV-Induced Gene 3 (EBI3) on the tumor microenvironment. An increase in EBI3 was detected in human CRC specimens and associated with phosphorylated STAT3. The administration of an EBI3 blocking peptide led to an antitumor cytotoxic T lymphocyte response and the inhibition of CRC cell proliferation in a tumor xenograft model [194]. In conclusion, although certain data may indicate an indirect role of EBV in CRC, any evidence beyond a possible association has not yet been proven.

#### 3.7.4. Cytomegalovirus

Another potentially important player in CRC carcinogenesis may be human CMV, which belongs to the Herpesviridae family of viruses. CMV has been shown to preferentially infect CRC lesions compared to normal healthy tissue [60,195,196]. Interestingly, a difference in CMV positivity was observed between Vietnamese and Swedish patients, with the former being significantly more infected by CMV [60]. The prevalence of CMV infection in a given population seems to depend on the geographical location. Further studies supporting this assumption are, however, necessary. Chen and colleagues revealed that the presence of CMV in CRC tumors was associated with a worse outcome and an increased expression of interleukin-17 in elder patients [197], whereas a more favorable disease-free survival rate was reported in CMV-positive non-elderly (aged < 65 years) CRC patients, suggesting that there may be a possible age-dependent effect [61]. However, other studies argued against the involvement of CMV in CRC carcinogenesis [198,199].

Multiple mechanisms have been proposed to contribute to the potential carcinogenic effect of CMV in CRC. For instance, it seems that genetic polymorphisms in the CMV genome may affect the outcome of CRC. Specifically, Chen et al. detected the presence of CMV in 47.8% of the CRC specimens analyzed. Among these, three different polymorphisms of the viral immunomodulatory gene UL144 (termed genotype A, B, and C) were identified. The presence of genotype B, occurring in 30.2% of the CMV-positive samples and characterized by the highest UL144 expression, was associated with shorter disease-free survival and independently predicted tumor recurrence [62]. Other discoveries came from studying the association between CMV and the expression of TLRs. In CMV-infected CRC and adenoma samples, the levels of expression of TLR2, TLR4, NF-κB, and TNF-α were higher compared to control tissues. These results were partially confirmed in the CRC cell line SW480, where only levels of TLR2 and TNF-α were increased [200]. In Caco-2 cells, CMV infection induced the expression of Bcl-2 and cyclooxygenase-2 (cox-2), which are two proteins involved in CRC carcinogenesis [201,202]. Interestingly, a study by Teo et al. showed that CMV infection promoted migration of the CRC cell lines HT29 and SW480. Mechanistically, this effect was associated with a six-fold upregulation of the Wnt/β-catenin signaling pathway compared to the non-infected cells [203]. Cai and co-workers investigated the role of the CMV-encoded US28 gene, coding one of the four G protein-coupled receptors believed to be essential for viral latent infection [204], in CRC pathogenesis [205]. The authors found a higher level of the US28 gene in CRC samples compared to adjacent normal tissue. Higher amounts of US28 were associated with histological grade, metastasis, Dukes’ stage, and survival [205]. In this context, US28-mediated activation of the Wnt/β-catenin pathway in CMV-infected cells has already been observed by Langemeijer and colleagues [206]. In addition, it has been shown that CMV may promote angiogenesis and carcinogenesis via the action of US28 and cox-2 [207]. Taken together, the available evidence strongly suggests that CMV may be able to interact with the mechanisms involved in CRC cell proliferation and progression. However, whether CMV can cause cell transformation by itself or should be viewed as a factor contributing to CRC development remains to be elucidated.

#### 3.7.5. John Cunningham Virus

Human polyomavirus 2, more commonly known as John Cunningham virus (JCV), which is a member of Polyomaviruses named after a patient with progressive multifocal leukoencephalopathy from whom it was isolated [208], has also been studied in relation to CRC [209]. In 1999, Laghi and colleagues firstly identified JCV in CRC colonic mucosa and suggested a possible link between the occurrence of JCV and CRC [52]. Since then, a number of studies have attempted to assess the prevalence of and, more recently, the association between, JCV and CRC. The majority of such studies point towards a possible carcinogenic role of JCV [53,54,210,211]. However, some reports did not find any significant difference between the presence or absence of JCV in CRC samples and thus concluded that the effect of JCV on CRC carcinogenesis either needed further validation or was non-existent [212,213,214,215].

**Figure 2:**
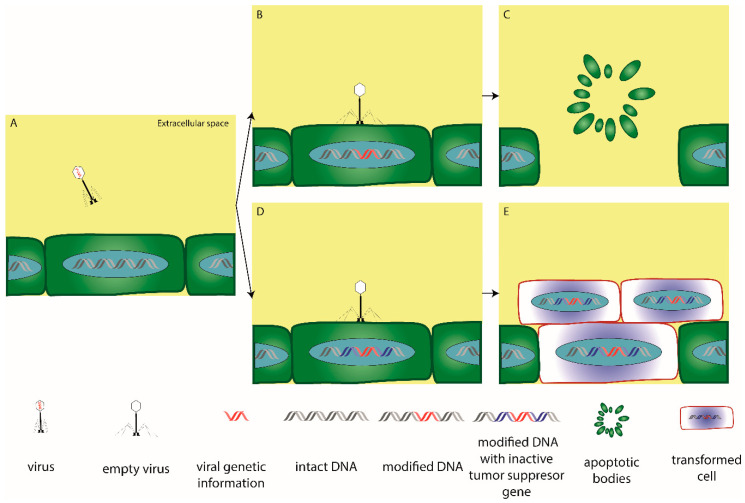
Example of virus-mediated carcinogenesis. A virus can infect a eukaryotic cell (**A**) and randomly insert its genetic information into the host genome via the lysogenic life cycle (**B**,**D**). If the viral genetic material falls in a non-coding region, the modified host DNA continues to function normally until the cell reaches senescence and is removed by apoptosis (**C**). However, the integration of the viral genome into a region coding a tumor suppressor gene (such as p53 or Rb), can affect cell proliferation/survival and, in combination with other DNA modifications, contribute to uncontrollable cell division, which may result in dysplasia and eventually, cancer (**E**).

JCV has been proposed to promote colon carcinogenesis in a number of ways. Firstly, a possible link between the expression of T-antigen (T-ag)—an early JCV protein which is believed to mediate the oncogenic potential of the virus—and chromosomal instability has been found [216,217]. In this regard, the hypermethylation of hMLH1 was associated with tumor JCV positivity and this suggested that the virus was capable of inducing aberrant methylation, as well as chromosomal instability [218]. Although the higher incidence of T-ag in patients with a family history of CRC did not correlate with mutations in DNA repair genes [219], these observations merit additional research. Secondly, JCV may be responsible for the induction of polymorphisms and/or alterations in tumor suppressor genes, such as p53 [220] (Figure 2), as well as for prioritizing for infection those cells which carry defects in tumor suppressor genes. This assumption is supported by the fact that p53 has been shown to inhibit JCV DNA replication via interacting with the T-ag. The highly conserved central region of p53 is responsible for such inhibition and this means that mutations in this region may affect the protection conferred by p53 [221]. Thirdly, JCV may alter cell behavior. After infecting with JCV the CRC cell lines HCT116 and SW837, cells expressing T-ag showed a two- to three-fold increase in migration and invasion compared to controls. Treatment with inhibitors of PI3K/AKT and MAPK pathways reduced both cell migration and invasion, thus underlining a possible involvement of such pathways in the JCV-driven increase of CRC metastasis [222]. Evidence supporting the involvement of JCV in the Wnt/β-catenin signaling cascade is even stronger. Enam et al. found that the coproduction of the viral T-ag and β-catenin in colon cancer cells enhanced the transcription of the oncogenic factor c-Myc [223]. Subsequently, the expression of TCF-4, which is a component of Wnt/β-catenin signaling, has been reported to decrease JCV DNA replication [224]. In this way, JCV may be able to interact with and modulate the Wnt pathway in colonic cells via the T-ag. These results were further confirmed by Ripple and colleagues, who found that the simultaneous presence of T-ag and β-catenin led to the activation of cell cycle regulators (i.e., c-Myc and cyclin D1) [225]. In another study, JCV was associated with p53, cyclin D1, and a family history of CRC, but neither with microsatellite instability nor changes in β-catenin and cox-2 expression [226].

While the body of evidence strongly supports a role for JCV in contributing to CRC carcinogenesis, future studies should be aimed at investigating the conditions under which such cell signaling regulation can happen.

### 3.8. Anal Cancer

While colon and rectal cancers share similar features and are often referred to collectively as CRC, anal cancer (AC) differs in significant ways, including the tissue where neoplastic transformation begins (similar to that lining the woman’s cervix), pathogenesis, and treatment [227]. Although AC is an uncommon tumor, accounting for about 2% of gastrointestinal malignancies, its frequency is increasing, especially in high-risk groups [228]. The predominant type of tumor (corresponding to 80% of all cases) is squamous cell carcinoma, which shares many features with cervical cancer [227]. Given the affinities with this latter tumor and the well-accepted role of HPV in cervical cancer pathogenesis, it is not surprising that the available evidence suggests a major role for this virus (in particular, the HPV-16) in the development of AC. In this regard, there are even estimates stating that HPV may be the underlying cause for as many as 90% of all AC cases [229]. Studies from different countries have reported the presence of HPV DNA in more than half of the AC specimens analyzed. For instance, Alexandrou et al. detected HPV DNA in 54.5% of tumor samples taken from Greek AC patients [43]. A Brazilian study reported an even higher HPV prevalence (69%), although it needs to be noted that HPV did not influence patient survival [230]. Using the United States National Cancer Database registry, Kabarriti et al. showed that 59.4% of non-metastatic AC patients treated with curative intent between 2008 and 2014 had HPV-positive disease [44]. Patients with an early stage of disease did not show significant differences in overall survival when stratified for HPV positivity. Surprisingly, in patients with HPV-positive advanced AC, the overall survival was better compared to HPV-negative patients with an equal disease stage [44]. In a recent observational, retrospective study carried out in North Sardinia, Italy, Muresu et al. assessed the prevalence and distribution of HPV genotypes in patients diagnosed with AC [45]. The overall HPV positivity was 70%, with HPV-16 being the predominant genotype (approximately 85%). The authors also found overexpression of the tumor suppressor protein p16^INK4a^, which is a surrogate marker for HPV infection that appears to have prognostic value in AC [231]. Despite this, the authors did not report any statistically significant difference in the overall survival between HPV-positive and -negative individuals, probably due to study limitations related to both sample size and epidemiological variables [45]. In contrast to this latter finding, when p16^INK4a^ was expressed in HPV-positive AC patients, better overall survival was observed [232,233] and at the same time, HPV-negative patients without p16 expression had a worse outcome [234]. Of particular importance is a study by Małusecka et al., who measured the HPV-16 viral load and expression of p16^INK4a^ and p53 in AC patients. The authors described an inverse trend in the association between the viral load and p16^INK4a^ expression status [235]. In addition to studies on human AC samples, HPV mouse models of anal carcinogenesis have been employed. For instance, one research group found that HPV-driven anal carcinogenesis can be modulated via autophagy. Their results showed that the systemic and topical administration of BEZ235, which is a PI3K/mTOR inhibitor, caused lower anal carcinogenesis via autophagy activation [236,237]. In 2018, Wechsler et al. firstly established an HPV-16 immortalized anal epithelial cell line (i.e., AKC2), which expressed the HPV genes E5, E6, and E7 [238]. These HPV genes have been associated with AC [238,239,240]. Upon a reduction of the E5 expression via short interfering RNA, lower amounts of both total and phosphorylated epidermal growth factor receptors were observed, along with reduced invasion. At the same time, the expression of E6 and E7 remained unaffected, thus supporting the role of E5 in the development of AC [238].

Apart from acting alone, HPV may cooperate with human immunodeficiency virus (HIV) to increase the probability of AC development. This is not surprising, as HIV patients bear a weakened immune system and thus have a higher chance of developing malignancies compared to the general population [241]. Studies have found a higher prevalence of AC in HIV patients [63], mainly in men who have sex with men [64,242]. In addition, Grew et al. observed significantly worse overall and colostomy-free survival rates in HIV-positive AC patients compared to HIV-negative ones [65]. Wentzensen et al. analyzed various biomarkers, such as HPV DNA, p16, and E6/E7 mRNA, in relation to AC severity in HIV-infected men who had sex with men. An increase in the above-mentioned biomarkers correlated with an increased severity of the disease, with HPV DNA having the highest sensitivity for anal intraepithelial dysplasia of grade 2 and 3 [243]. Another study suggested a possibility for HPV-16 E6 serology, despite its low sensitivity, to characterize a group of individuals with a very high anal cancer incidence and predict AC risk [244]. Bertisch and colleagues reported that, apart from HPV E6, the HPV L1 gene and smoking were significantly associated with AC [245]. Even though a study dated to 2013 showed that the recurrence rate of AC was associated with HIV positivity, but not with HPV/p16 [246], Liu and colleagues recently reported an association between HPV-16 positivity and the presence of high-grade lesions in AC patients [247]. In conclusion, accumulating evidence indicates that HPV infection, either alone or concomitant with HIV infection, likely contributes to the development of AC.

## 4. Discussion and Future Outlooks

The study of microbial entities that may be involved in the development of malignancies of the alimentary tract has mainly concentrated on the bacterial component of the microbiome [2]. Another large, but less studied, portion of the digestive system microbiome is that consisting of both bacteriophages and viruses (infecting prokaryotic and eukaryotic cells, respectively), together known as the virome [3]. Despite the recent advent of cutting-edge technologies in nucleic acid sequencing, omics analysis, and bioinformatics pipelines, characterization of the gut virome and its role in the development of pathological conditions has been slowed by several hurdles and is still in the early stages.

Most of the studies described herein have focused on the prevalence of infection with a certain virus in a type of cancer in a given geographical location. While data gathered in such a manner can be undoubtedly seen as a necessary first step, it is important to point out that such studies do not carry the collective knowledge beyond the level of association and may be hampered by methodological flaws. Indeed, research on patients usually contains many potentially confounding factors (e.g., age, sex, environment, geography, lifestyle and sexual habits, and simultaneous comorbidities) that may reasonably impact the structure and function of the virome in the short or long term. The use of related individuals living in the same household/environment, rather than unrelated randomly selected individuals, may be useful for reducing such a bias. Another concern relates to cross-study comparisons of datasets, which are almost impossible due to differences in sample collection, handling, storage, and processing (e.g., isolation of viral-like particles, DNA/RNA extraction, sequencing platforms, and parameters). The development of standardized protocols, as well as the establishment of Gold Standard procedures, would help address this issue. Finally, as the virome composition may differ from early to late stages of disease, research in this area should take into account a rigorous analysis of tumor stadiation. Discovering molecular mechanisms behind virus–cell communication may be of particular importance in the future. To elucidate the potential carcinogenic role of a given virus, functional studies involving cell cultures and animal models should be employed to a greater extent. However, determining the functional impact of a virus as a cancer agent requires effective vaccines or antivirals. If eliminating the infection also eliminates the cancer, then the link is clear. Unfortunately, such an approach requires specific drug and vaccine development, which only occurs once a virus is already established to cause a disease or cancer, and there is sufficient economic interest on the part of biopharmaceutical companies. In 2018, about 2.2 million cancer cases, or 13% of the global cancer incidence, were attributable to infection (and in particular, to viruses) [248]. In accordance with this assumption, a number of cancers of the digestive system have either been proven or are believed to come from a relatively small number of viral strains (i.e., HBV, HCV, and HPV). This means that a fraction of such cancers could be, in theory, preventable by vaccination. As preventive vaccines for HBV and HPV are fortunately available, it should be one of the priorities of governments to educate people and provide measures (i.e., HBV and HPV prophylaxis), in order to minimize the carcinogenic impact of such viral infections and subsequently lower the cancer burden on the healthcare system. 

A sector of the virome that remains inadequately investigated is the phageome. This is particularly true for the colon, which harbors conditions such as a mild pH and nutrient-rich environment, allowing for the abundant occurrence of bacteria and thus phages. Although research in the field has made great strides, whether phages play a role in the development of gastrointestinal cancer remains an unanswered question. To date, publications considering the possibility that phages may be a driving force of carcinogenesis in the gut are lacking [249]. Certain phages could theoretically act as mediators of carcinogenesis, for example, due to endowing bacteria with genes by horizontal gene transfer. Conversely, other phages may provide a protective effect, preying on those bacteria with increased invasion and fitness [20]. One of key tasks of future virome investigations should be to elucidate which phages and circumstances could tip the balance towards either a carcinogenic or anti-carcinogenic effect.

While some of the potential virome-mediated carcinogenic effects occurring in the gut can be induced by a direct interaction of viruses with the host cells, others may require the interplay of viruses with other components of the microbiome (commensal bacteria, archaea, fungi, and protozoans, among other organisms). The above-mentioned interactions may take place regardless of whether the enteric viruses replicate locally in the intestine or whether they use the gut as a point of entry to the host, showing their pathologic effects in organs other than the gastrointestinal tract [30]. To help unravel these complex connections, future gut virome research should be complemented by that on gut bacterial and fungal diversity.

Notwithstanding, it is important to point out that viral infections are generally not sufficient for cancer development, as cancers are evolutionarily dead-end events that threaten the viral agent as much as the host. Indeed, viral-mediated tumors only occur in a minor fraction of infected individuals when additional events (e.g., immune system suppression and exposure to carcinogens) and/or concomitant host factors (e.g., genetic predisposition and somatic mutations) exist.

## 5. Conclusions

Despite the recent increase of the awareness about the relationship between specific viruses (i.e., HBV, HCV, EBV, and JCV) and the pathogenesis of oral, hepatic, colorectal, and anal cancers, the human virome remains largely uncharacterized and we still have a poor knowledge on how it may influence the risk of developing cancers of the digestive system. Further mechanistic studies using experimental models and multicenter observational cohort studies—designed to pay attention to the potential biases—would hopefully help with further unravelling such a complex topic.

## Figures and Tables

**Table 1:** Overview of the most relevant studies investigating potential associations between viruses and cancers of the digestive system.

First Author	Year	Virus	Cancer Type	Sample Origin	Number of Samples	Observation	Ref.
Maden	1992	HPV	Oral	USA	131	HPV-6 is associated with oral cancer	[36]
Jalouli	2012	HPV, HSV, EBV	Oral	UK, Sweden, Sudan, Norway, USA, Yemen, India, Sri Lanka	155	Higher proportion of samples with HSV and EBV in industrialized countries	[37]
Bjørge	1997	HPV	Esophageal	Norway	228	Increased risk of developing cancer among HPV-16-seropositive subjects	[38]
Zhang	2015	HPV	Esophageal	China	3044	HPV-16 is a possible risk factor	[39]
Wang	2010	HPV	Esophageal	China, USA	435	HPV infection is common in esophageal carcinoma, independent of region and ethnic group of origin	[40]
Kirgan	1990	HPV	Colorectal	USA	90	Association between HPV and colon neoplasia	[41]
Lee	2001	HPV	Colorectal	Taiwan	38	HPV-18 is a possible risk factor	[42]
Alexandrou	2014	HPV	Anal	Greece	11	Lower incidence of HPV in anal cancer compared to other Western countries	[43]
Kabarriti	2019	HPV	Anal	USA	5927	HPV is a significant prognostic marker in anal cancer, especially for locally advanced disease	[44]
Muresu	2020	HPV	Anal	Italy	30	HPV is a possible risk factor	[45]
Awerkiew	2003	HPV, EBV	Esophageal	Germany	37	EBV, but not HPV, was detected in esophageal cancer samples	[46]
Martínez-López	2014	EBV	Gastric	Mexico	297	Possible role for EBV in gastric cancer and early precursor lesions.	[47]
Corallo	2020	EBV	Gastric	Italy	175	Patients with EBV-positive gastric cancer who did not receive ICI had a better response to first-line chemotherapy and better survival compared with EBV-negative patients	[48]
Li	2004	EBV	Hepatic	China	141	Presence of HBV infection in HCC tissues	[49]
Song	2006	EBV	Colorectal	China	115	Possible association of EBV with colorectal carcinoma	[50]
Fiorina	2014	HPV, EBV, JCV, BKV	Colorectal	Italy	44	No or weak association of HPV, EBV, JCV, and BKV with colorectal cancer	[51]
Laghi	1999	JCV	Colorectal	USA	23	JCV DNA may play a role in the chromosomal instability observed in colorectal carcinogenesis	[52]
Hori	2005	JCV	Colorectal	Japan	64	Possible role of JCV in colorectal carcinogenesis	[53]
Jung	2008	JCV	Colorectal	USA	74	JCV T-Antigen is expressed in the early stage of colorectal cancer	[54]
Tokita	2002	TTV	Hepatic	Japan	237	High TTV abundance is an independent risk factor	[55]
Iloeje	2010	HBV	Pancreatic	Taiwan	22,471	Chronic HBV infection may be associated with an increased risk of pancreatic cancer	[56]
Zhou	2012	HBV, HCV	Bile duct	Meta-analyses (13 case-control studies and 3 cohort studies)	-	HBV and HCV are risk factors in bile duct cancer	[57]
Hassan	2008	HBV	Pancreatic	USA	476	Past exposure to HBV is a possible risk factor in pancreatic cancer	[58]
Su	2020	HBV	Colorectal	Taiwan	69,478	Chronic HBV infection is strongly associated with increased risk of developing colorectal cancer	[59]
Dimberg	2013	CMV	Colorectal	Sweden, Vietnam	202	CMV DNA rate was significantly higher in cancerous tissues compared to normal tissues	[60]
Chen	2016	CMV	Colorectal	Taiwan	556	More favorable disease-free survival rate in non-elderly patients with CMV-positive tumors, specifically in patients with stage III disease	[61]
Chen	2015	CMV	Colorectal	USA, France, Italy, Japan, China, Taiwan	92	Specific genetic CMV polymorphisms are associated with different clinical outcomes	[62]
Jin	2019	HIV	Anal	Australia	Not applicable	People living with HIV are at markedly higher risk of anal cancer	[63]
Colón-López	2018	HIV	Anal	USA	Not applicable	Anal cancer incidence is markedly elevated among people with HIV infection	[64]
Grew	2015	HIV	Anal	USA	Not applicable	HIV-positive patients had significantly worse overall and colostomy-free survival rates than HIV-negative patients	[65]

Abbreviations: HPV: human papillomavirus; HSV: herpes simplex virus; EBV: Epstein–Barr virus; ICI: immune-checkpoint inhibitor; HCC: hepatocellular carcinoma; JCV: John Cunningham virus; BKV: BK virus; TTV: TT virus; HBV: hepatitis B virus; HCV: hepatitis C virus; CMV: cytomegalovirus; HIV: human immunodeficiency virus. Not applicable means that the study did not involve human specimens.
